# IL-1R2-based biomarker models predict melioidosis mortality independent of clinical data

**DOI:** 10.3389/fmed.2023.1211265

**Published:** 2023-06-29

**Authors:** Taniya Kaewarpai, Shelton W. Wright, Thatcha Yimthin, Rungnapa Phunpang, Adul Dulsuk, Lara Lovelace-Macon, Guilhem F. Rerolle, Denisse B. Dow, Viriya Hantrakun, Nicholas P. J. Day, Ganjana Lertmemongkolchai, Direk Limmathurotsakul, T. Eoin West, Narisara Chantratita

**Affiliations:** ^1^Department of Microbiology and Immunology, Faculty of Tropical Medicine, Mahidol University, Bangkok, Thailand; ^2^Division of Pediatric Critical Care Medicine, Department of Pediatrics, University of Washington, Seattle, WA, United States; ^3^Mahidol-Oxford Tropical Medicine Research Unit, Faculty of Tropical Medicine, Mahidol University, Bangkok, Thailand; ^4^Division of Pulmonary, Critical Care and Sleep Medicine, Harborview Medical Center, University of Washington, Seattle, WA, United States; ^5^Center of Tropical Medicine and Global Health, University of Oxford, Oxford, United Kingdom; ^6^Cellular and Molecular Immunology Unit, Centre for Research and Development of Medical Diagnostic Laboratories, Faculty of Associated Medical Sciences, Khon Kaen University, Khon Kaen, Thailand; ^7^Department of Medical Technology, Faculty of Associated Medical Science, Chiang Mai University, Chiang Mai, Thailand; ^8^Department of Tropical Hygiene, Faculty of Tropical Medicine, Mahidol University, Bangkok, Thailand

**Keywords:** biomarkers, melioidosis, IL-1R2, sTREM-1, mortality

## Abstract

**Introduction:**

Melioidosis is an often-fatal tropical infectious disease caused by the Gram-negative bacillus *Burkholderia pseudomallei*, but few studies have identified promising biomarker candidates to predict outcome.

**Methods:**

In 78 prospectively enrolled patients hospitalized with melioidosis, six candidate protein biomarkers, identified from the literature, were measured in plasma at enrollment. A multi-biomarker model was developed using least absolute shrinkage and selection operator (LASSO) regression, and mortality discrimination was compared to a clinical variable model by receiver operating characteristic curve analysis. Mortality prediction was confirmed in an external validation set of 191 prospectively enrolled patients hospitalized with melioidosis.

**Results:**

LASSO regression selected IL-1R2 and soluble triggering receptor on myeloid cells 1 (sTREM-1) for inclusion in the candidate biomarker model. The areas under the receiver operating characteristic curve (AUC) for mortality discrimination for the IL-1R2 + sTREM-1 model (AUC 0.81, 95% CI 0.72–0.91) as well as for an IL-1R2-only model (AUC 0.78, 95% CI 0.68–0.88) were higher than for a model based on a modified Sequential Organ Failure Assessment (SOFA) score (AUC 0.69, 95% CI 0.56–0.81, *p* < 0.01, *p* = 0.03, respectively). In the external validation set, the IL-1R2 + sTREM-1 model (AUC 0.86, 95% CI 0.81–0.92) had superior 28-day mortality discrimination compared to a modified SOFA model (AUC 0.80, 95% CI 0.74–0.86, *p* < 0.01) and was similar to a model containing IL-1R2 alone (AUC 0.82, 95% CI 0.76–0.88, *p* = 0.33).

**Conclusion:**

Biomarker models containing IL-1R2 had improved 28-day mortality prediction compared to clinical variable models in melioidosis and may be targets for future, rapid test development.

## Introduction

Melioidosis is a severe infectious disease caused by the environmental saprophyte *Burkholderia pseudomallei*. Melioidosis is endemic to parts of Southeast Asia, South Asia, Northern Australia with sporadic cases reported in the Americas (1). The clinical manifestations of melioidosis are often variable but commonly include pneumonia, bacteremia, abscesses, and sepsis. The clinical course of melioidosis is also unpredictable, though patients frequently develop multi-organ dysfunction leading to death (2). Despite appropriate antibiotic treatment, the mortality rate from melioidosis can exceed 40% in Southeast Asia (3).

Identifying patients at highest risk of poor outcomes can help in guiding clinical management and resource allocation. Clinical severity illness scores–such as the Sequential Organ Failure Assessment (SOFA) and the Acute Physiology and Chronic Health Evaluation (APACHE) III score–reasonably predict mortality in patients with sepsis in Southeast Asia; however, these approaches require extensive clinical and laboratory data, limiting their utility in low- and middle-income countries (LMIC) where many melioidosis cases may occur (4–6). Many critically ill patients in LMIC are treated outside of intensive care settings, further limiting the utility of these scoring systems (7). Biomarkers, when integrated into clinical laboratory or point-of-care tests, are potential tools for rapid patient management and triage decisions, critical in LMIC where resources or information about baseline co-morbidities may be limited. We recently reported that the inclusion of IL-6 and IL-8 plasma concentrations augments a model comprised of clinical variables in predicting mortality in melioidosis (8). Elevated concentrations of other inflammatory biomarkers, including calprotectin (a heterodimer containing S100A9), C-reactive protein (CRP), procalcitonin (PCT) and soluble triggering receptor on myeloid cells 1 (sTREM-1) have been associated with worse outcome or lack of response to therapy in melioidosis (9–11). However, in patients suspected of melioidosis there is still a critical need for biomarkers which can reliably predict severe outcomes but do not require additional clinical variables and that can potentially be used to develop future point-of-care tests for use in resource-limited settings.

In the last several years, commercially-available tests have been developed for the rapid, bedside diagnosis of melioidosis (12). Additionally, we recently reported that melioidosis non-survivors have increased expression of gene associated with interleukin 1 receptor, type 2 (IL-1R2) in peripheral blood leukocytes compared to melioidosis survivors (13). Therefore, we sought to extend this bedside diagnostic approach to include prediction in patients already diagnosed with, or suspected of, melioidosis. We hypothesized that plasma concentrations of IL-1R2 protein could predict outcome similar to a model of clinical variables in patients with melioidosis. Additionally, we hypothesized that a multivariable model combining IL-1R2 and other non-correlated inflammatory protein biomarkers could surpass clinical prediction models in patients with melioidosis. We therefore performed an extensive literature review and identified five additional inflammatory biomarkers representing alternative inflammatory pathways which might provide complimentary outcome prediction to, or even surpass, IL-1R2. In this study, we then developed and tested these biomarker models to predict melioidosis-related mortality. We subsequently validated the strongest prediction models in an external cohort of patients with melioidosis.

## Methods

### Patient cohorts

#### Derivation cohort

Subjects aged 15 years or older admitted to Udon Thani Hospital, Udon Thani, Thailand from 2015 to 2018 were prospectively enrolled within 24 h of *B. pseudomallei* culture positivity. This cohort has previously been described in part (8, 13). Plasma samples were drawn at enrollment. Clinical information was abstracted from the medical records or determined by the study team and reported in a case report form. Mortality at 28 days from the time of enrollment was obtained from medical records or patient interviews by telephone. Ninety patients with available plasma were selected for plasma biomarker measurement, and 78 subjects with complete biomarker data and an enrollment time of less than 7 days after admission were included in the final analysis.

#### External validation cohort

Subjects aged 18 years or older who were admitted from 2013 to 2017 to Sunpasittiprasong Hospital, Ubon Ratchathani, Thailand with suspected infection were prospectively enrolled. Enrollment occurred within 24 h of admission to the hospital. This cohort as well as subsets have been previously reported (7, 14). Plasma samples were obtained at enrollment, and clinical data and 28-day mortality were obtained from medical records and patient interviews. One hundred and ninety-one enrolled patients had a specimen that was culture-positive for *B. pseudomallei* and so were included in the external validation cohort.

### Biomarker concentration measurement

In the derivation cohort, six biomarkers were measured by ELISA: IL-1R2, CRP, PCT, sTREM-1, calprotectin (Abcam) as well as S100A9 (Biolegend). In the external validation cohort, plasma concentrations of IL-1R2 (Abcam) and sTREM-1 (R&D Systems) were measured by ELISA. Samples were tested and analyzed per the manufacturers’ instructions.

### Clinical definitions

The SOFA score was originally designed as a sequential scoring system using clinical and laboratory data as markers of organ dysfunction and/or failure, including respiratory, coagulation, hepatic, cardiovascular and renal components (4). In the derivation cohort, an organ failure score was generated using clinical data from within the first 24 h of admission by modifying a SOFA score. These modifications were necessary because clinical data obtained at enrollment lacked a mental status assessment, arterial blood gas measurements, or vasoactive drug doses necessary to calculate a complete SOFA score. The organ failure score was calculated using the respiratory, cardiovascular, liver, renal, and coagulation measurements from a SOFA score. For the respiratory component of the score, 2 points were given to patients requiring mechanical ventilation when no arterial blood gas was available. For the cardiovascular component of the score, 2 points were given to patients requiring dobutamine or dopamine and 3 points were given to patients requiring norepinephrine or epinephrine.

In the external validation cohort, a modified SOFA score including the Glasgow Coma Scale (GCS) was calculated by adding the highest component score from the time of presentation to the time of study enrollment, which was within 24 h of admission. Because arterial blood gases are not frequently used at the study hospital and dosages of vasoactive medications were not recorded, the cardiovascular and respiratory components of the SOFA score were modified as described above and as previously reported (14).

### Model development and statistical analysis

Novel candidate models of mortality prediction were developed according to Transparent Reporting of a Multivariable Prediction Model for Individual Prognosis or Diagnosis (TRIPOD) recommendations (15). To develop a potentially multibiomarker-based prediction model, as recommended by TRIPOD guidelines, to reduce bias and to develop the simplest multivariable prediction model, we employed a least absolute shrinkage and selection operator (LASSO) approach to select the biomarkers to include in a final multivariable model (16). LASSO minimizes bias in multivariable model selection by employing shrinkage penalties on each included variable coefficient and then sequentially eliminating variables, including those with high correlation to other variables. Therefore, all measured biomarkers, regardless of their association with mortality, were included in the LASSO selection. Lambda was selected by Bayesian Information Criterion, and the selected biomarkers were confirmed when using the largest lambda within one standard error of the minimal mean squared prediction error based on 10-fold internal cross-validation analysis (17). Biomarkers chosen by LASSO were then included in a candidate multivariable prediction model. Twenty-eight-day mortality prediction of LASSO-selected biomarkers was evaluated using logistic regression models. Models were assessed for calibration by Hosmer-Lemeshow goodness-of-fit chi-square analysis and bias by internal validation of 1,000 replication sets by bootstrapping (18, 19). Mortality discrimination was determined by generating receiver operating characteristic curves and calculating the area under the curve. To assess the improvement of model performance after the addition of individual biomarkers, the likelihood ratio test and integrated improvement analysis (IDI) were used (20, 21). To assess clinical utility, the net benefit of selected models across a range of risk thresholds was compared using decision curve analysis (22). For the final model, an optimal cut-off value representing the optimal discrimination was determined using a Youden’s index (23). Subsequently, the sensitivity, specificity, negative and positive predictive likelihood ratios with corresponding 95% confidence intervals were calculated.

Variables were reported as median and interquartile range (IQR) for non-normally distributed data. Differences in biomarker concentrations were evaluated by the Mann–Whitney U test. Biomarkers were log10-transformed prior to regression analysis and the association of selected biomarkers with 28-day mortality was determined by logistic regression.

Correlations between biomarkers were assessed using a Spearman’s rank-order correlation. Mortality prediction models of clinical variables were generated using the cohort-specific organ failure score or modified SOFA score. Analyzes were performed using Stata SE version 14.2 (StataCorp, College Station, TX, United States).

## Results

### Derivation cohort clinical characteristics

Plasma samples were obtained at study enrollment from patients with culture-proven melioidosis in the derivation cohort. The study flow diagram is shown in Supplementary Figure S1, and the characteristics of this cohort are shown in Table 1. The median age was 58 years (IQR, 47–67 years) and 77% were male. The median Charlson Comorbidity Index was 3 (IQR 1–4) and 72% of patients had diabetes. The median time to enrollment in this cohort was 4 days and 37% of the patients died within 28 days.

**Table 1:** Clinical characteristics of melioidosis patients.

Characteristics	Derivation cohort (*N* = 78)	External validation cohort (*N* = 191)
Demographics		
Age in years, median (IQR)	58 (48–68)	54 (46–64)
Male sex, *N* (%)	60 (77)	132 (69)
Pre-existing conditions		
Charlson Comorbidity Index, median (IQR)	3 (2–5)	2 (1–3)
Diabetes, *N* (%)	56 (72)	88 (46)
Chronic kidney disease, *N* (%)	20 (26)	26 (14)
Chronic lung disease, *N* (%)	7 (9)	15 (8)
Chronic liver disease, *N* (%)	2 (3)	7 (4)
Chronic cardiovascular disease, *N* (%)	6 (8)	11 (6)
Cancer, *N* (%)	5 (6)	3 (2)
Human immunodeficiency virus, *N* (%)	2 (3)	0 (0)
Days to enrollment, median (IQR)	4 (3–5)	1 (0–1)
Organ failure score^a^	2 (0–5)	-
Modified SOFA score, median (IQR)	-	5 (2–9)
Died within 28 days, *N* (%)	29 (37)	98 (51)

a Organ failure score was derived from the available components of a SOFA score using clinical and laboratory data within the first 24 h of admission.

### Plasma biomarkers are associated with 28-day mortality in melioidosis

Plasma concentrations of IL-1R2, CRP, PCT, sTREM-1, S100A9 and calprotectin were measured at the time of study enrollment and were compared between survivors and non-survivors at 28-days. Concentrations of IL-1R2 and PCT were significantly higher in non-survivors compared to survivors (both *p* < 0.01; Table 2). We tested the association of each of the six log10-transformed biomarkers with 28-day mortality. IL-1R2 was the only measured biomarker significantly associated with 28-day mortality when adjusting for baseline risk factors including age, sex and Charlson Comorbidity Index plus organ failure score (OR 210.8, 95% CI 13.4–3,381, *p* < 0.001; Table 3).

**Table 2:** Biomarkers by 28-day survival status of melioidosis patients.

Biomarker (pg/ml): median (IQR)	All (*N* = 78)	Survivors (*N* = 49)	Non-survivors (*N* = 29)	*P* value
IL-1R2	116,823 (52,553-238,368)	59,429 (38,175-147,727)	199,846 (128,661-274,000)	<0.001
CRP	631,247 (491,097-722,272)	629,802 (482,428-690,485)	638,471 (508,435-730,941)	0.45
PCT	1841 (913–2025)	1,677 (697–1983)	1966 (1824–2037)	0.004
sTREM-1	200 (137–440)	187 (112–301)	238 (187–491)	0.05
S100A9	3,206 (2575–4,044)	3,023 (2327–3,720)	3,673 (2652–4,230)	0.10
Calprotectin	1.5×10^7^ (9.2 ×10^6^-2.3×10^7^)	1.2×10^7^ (8.2×10^6^-2.4×10^7^)	1.7×10^7^ (1×10^7^-2.2×10^7^)	0.22

**Table 3:** Association of biomarkers with 28-day mortality in the derivation cohort.

Biomarker (log_10_)	Unadjusted			Baseline-adjusted^a^			Baseline + organ failure-adjusted^b^		
	OR	95% CI	*P* value	OR	95% CI	*P* value	OR	95% CI	*P* value
IL-1R2	33.4	5.8–192.8	<0.001	43.0	8.2–224.9	<0.001	210.8	13.4–3,381	<0.001
CRP	2.7	0.3–24.9	0.37	3.3	0.4–29.4	0.3	2.0	0.2–19.8	0.57
PCT	80.4	2.5–2565.4	0.01	72.4	0.8–6964.8	0.07	72.3	0.5–9,703	0.09
sTREM-1	3.5	1.0–12.2	0.06	2.9	0.9–9.5	0.09	2.7	0.9–8.7	0.09
S100A9	4.2	0.4–40.8	0.22	6.1	0.5–71.9	0.15	5.6	0.5–66.1	0.17
Calprotectin	2.8	0.6–11.9	0.17	3.1	0.7–13.9	0.14	2.3	0.5–10.5	0.27

a Adjusted for age, sex, and Charlson Comorbidity Index.

b Adjusted for age, sex, Charlson Comorbidity Index, and organ failure score.

### Model development

To develop the simplest yet unbiased mortality prediction model, we performed LASSO regression on all six measured biomarkers from the derivation cohort, regardless of association with 28-day mortality. LASSO regression selected IL-1R2 and sTREM-1 for the final model. IL-1R2 and sTREM-1 were not significantly correlated (rho = 0.07, *p* = 0.53) and so both biomarkers were carried forward for further evaluation.

**Figure 1:**
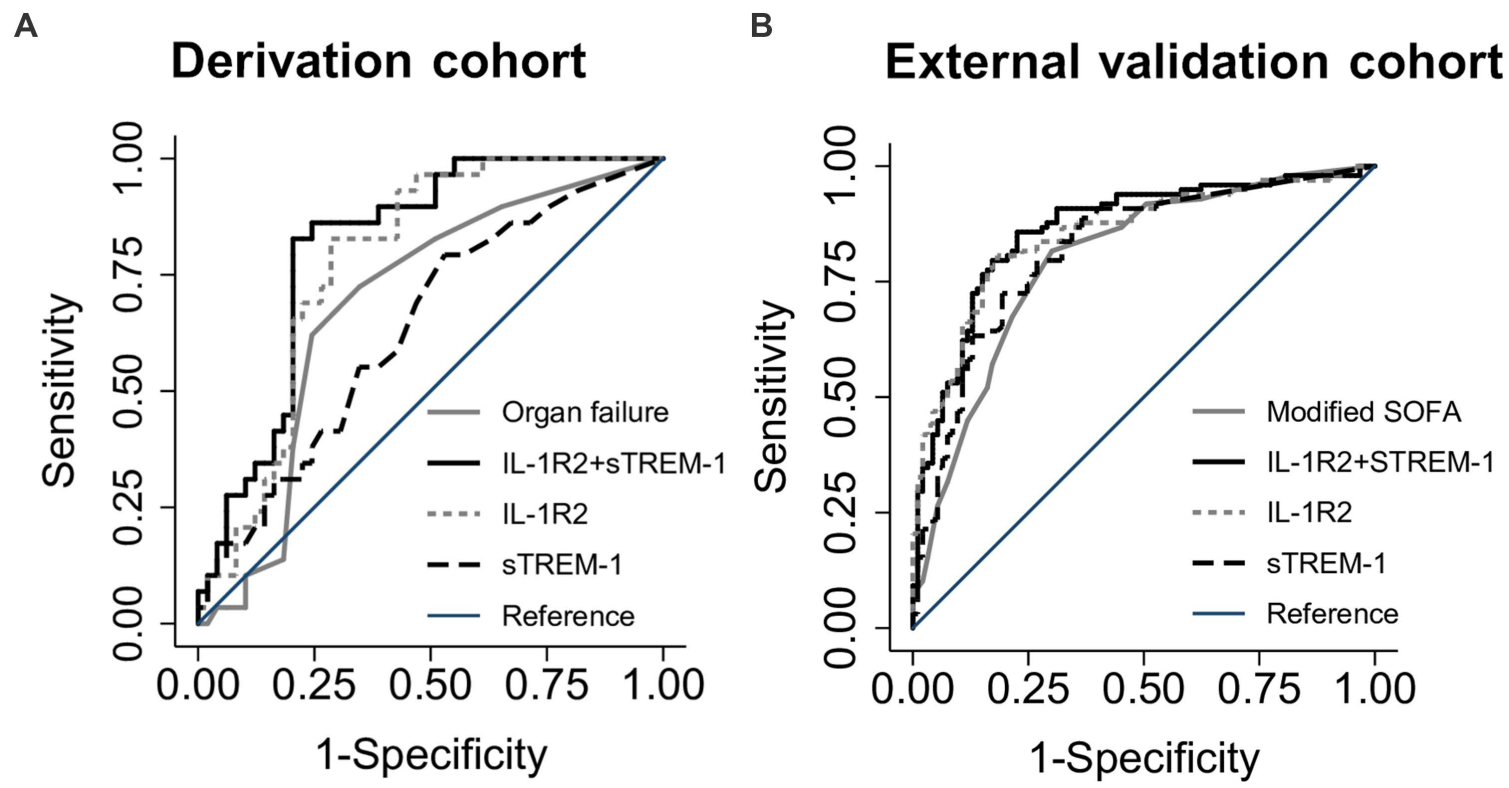
Receiver operating characteristic curves for discrimination of death at 28 days. Area under the receiver operating characteristic curves (AUC) for the two-biomarker model (IL-1R2 + sTREM-1) compared to IL-1R2 and sTREM-1 single biomarker models and an organ failure or modified SOFA score in either the derivation **(A)** or external validation **(B)** cohorts.

We next compared mortality discrimination of the two-biomarker model in comparison to a model comprised of the organ failure score using receiver operating characteristic curve analysis. The area under the receiver operating characteristic curve (AUC) was calculated for both models as well as single biomarker models containing IL-1R2 and sTREM-1 (Table 4; Figure 1A). The two-biomarker model had significantly higher mortality discrimination (AUC 0.81, 95% CI 0.72–0.91) compared to the organ failure score model (AUC 0.69, 95% CI 0.56–0.81; *p* < 0.01). Additionally, the IL-1R2 model had significantly higher mortality discrimination compared to the organ failure score model (AUC 0.78, 95% CI 0.68–0.88; *p* = 0.03) and similar discrimination compared to the two-biomarker model (*p* = 0.21). All models maintained their mortality discrimination after optimism-adjustment by bootstrapping and were assessed for calibration by goodness-of-fit testing (Supplementary Table S1).

**Table 4:** Mortality discrimination in the derivation and external validation cohorts.

Model	AUC (95% CI)	*P* value^b^
Derivation cohort		
Organ failure^a^	0.69 (0.56–0.81)	Ref
IL-1R2 + sTREM-1	0.81 (0.72–0.91)	<0.01
IL-1R2^c^	0.78 (0.68–0.88)	0.03
sTREM-1	0.64 (0.51–0.76)	0.56
External validation cohort		
Modified SOFA	0.80 (0.74–0.86)	Ref
IL-1R2 + sTREM-1	0.86 (0.81–0.92)	<0.01
IL-1R2^d^	0.85 (0.80–0.91)	0.06
sTREM-1	0.82 (0.76–0.88)	0.46

a Model consists of the organ failure score which was derived from the available components of a SOFA score using clinical and laboratory data within the first 24 h of admission.

b *P* value represents comparison of biomarker model to the cohort-specific organ failure or modified SOFA model.

c *P*-value of the IL-1R2 + sTREM-1 model compared to the IL-1R2 model = 0.21.

d *P*-value of the IL-1R2 + sTREM-1 model compared to the IL-1R2 model = 0.33.

Given the similar mortality discrimination of the two-biomarker model and the IL-1R2 model, we next tested whether the IL-1R2 model had improved performance after the addition of sTREM-1. The addition of sTREM-1 did significantly improve the IL-1R2 model (likelihood ratio (LR) *p* = 0.02; Supplementary Table S2) though discrimination was only borderline improved (IDI = 0.06, *p* = 0.05). Given these findings, both models were brought forward for validation in an external cohort.

### External cohort validation

We next sought to validate the IL-1R2 + sTREM-1 and IL-1R2 models in an external cohort of melioidosis patients (Table 1). This cohort was larger than the derivation cohort, enrolled subjects less than 24 h after study hospital presentation and captured sufficient data to calculate a modified SOFA score including GCS. In this cohort, concentrations of both IL-1R2 and sTREM-1 were significantly higher in non-survivors compared to survivors (both *p* < 0.001; Supplementary Table S3). IL-1R2 and sTREM-1 were also significantly associated with 28-day mortality in models adjusted for baseline factors including age, sex, Charlson Comorbidity Index, with and without modified SOFA score (all *p* < 0.001; Table 5). The two-biomarker model significantly improved mortality discrimination compared to a modified SOFA score model (AUC 0.86, 95% CI 0.81–0.92 vs. AUC 0.80, 95% CI 0.74–0.86, *p* < 0.01; Figure 1B; Table 4). The IL-1R2 model trended toward significantly higher discrimination compared to the modified SOFA model (AUC 0.85, 95% CI 0.80–0.91, *p* = 0.06) and was similar to the two-biomarker model (*p* = 0.33). Both models had unchanged discrimination after bias-adjustment and appropriate goodness-of-fit (Supplementary Table S1).

**Table 5:** Association of biomarkers with 28-day mortality in the external validation cohort.

Biomarker (log_10_)	Unadjusted			Baseline-adjusted^a^			Baseline + SOFA-adjusted^b^		
	OR	95% CI	*P* value	OR	95% CI	*P* value	OR	95% CI	*P* value
IL-1R2	35.9	13.8–93.3	<0.001	47.4	17.0–132.3	<0.001	28.2	7.8–101.7	<0.001
sTREM-1	18.4	8.0–42.0	<0.001	18.4	7.9–42.9	<0.001	8.1	3.0–22.3	<0.001

a Adjusted for age, sex, and Charlson Comorbidity Index.

b Adjusted for age, sex, Charlson Comorbidity Index, and modified SOFA score.

As the two-biomarker model had similar mortality discrimination to the IL-1R2 model, we next sought to determine if there was any clinical benefit of using the two-biomarker model compared to the IL-1R2 model by decision curve analysis. The IL-1R2 + sTREM-1 model had a higher clinical benefit for a cohort mortality of 10% to 50% (Supplementary Figure S2; Supplementary Table S4). However, the increase in correct identification of patients who would die in 28-days using the two-biomarker model was modest, ranging between 1 to 2.3 patients per 100.

As a one-biomarker model may have simplistic advantages and since the IL-1R2 model was nearly identical to the two-biomarker model in terms of mortality discrimination and clinical benefit, we next sought to calculate a reasonable prognostic cut-off value for IL-1R2. An optimal cut-off value of 173,911 pg/mL yielded a sensitivity for death of 72% (95% CI 63–81%) and specificity of 85% (95% CI 76–92%; Supplementary Table S5).

## Discussion

In this study, we report the development and validation of a plasma biomarker model to predict mortality in melioidosis. A model comprised of plasma concentrations of IL-1R2 and sTREM-1 performed better than an organ failure model in both our derivation and external validation cohorts in predicting mortality in melioidosis. Additionally, a simple model composed only of IL-1R2 performed nearly as well as the two-biomarker model and may be a reasonable substitute. Implemented for clinical use, these biomarker profiles may have value for melioidosis outcome prognostication in regions where the infection is endemic and may complement emerging rapid diagnostic tests (12).

We have previously reported that IL-6 and IL-8 concentrations augment the melioidosis mortality prediction of a clinical model (24). However, whether a biomarker model may be able to replace a clinical prediction model in melioidosis for prognostication has not been established. Clinical scores such as SOFA require multiple laboratory and clinical data, including the availability of mechanical ventilation and circulatory support. In our study, IL-1R2-containing models, which do not include any clinical variables, had superior performance compared to a comprehensive clinical variable model.

During systemic infection, IL-1R2 may serve as a decoy receptor for capturing and reducing the bioavailability of the pro-inflammatory cytokine IL-1 (25). Notably, some *in vivo* melioidosis models suggest IL-1 signaling may be detrimental. IL-1R1 knockout mice display increased resistance to *B. pseudomallei* infection but with decreased lung neutrophil recruitment and lower associated lung pathology (26). Similarly, anti-IL-1β treatment of *B. pseudomallei*-infected mice decreased bacterial replication and was associated with reduced mortality (27). In humans, up-regulated transcription of the IL1R2-associated gene has been described in critically ill patients with sepsis (28). Our novel finding that IL-1R2 plasma concentration is associated with mortality in melioidosis supports our recent report that blood leukocyte *IL1R2* upregulation is likewise associated with melioidosis mortality (13). Triggering receptor expressed on myeloid cells 1 (TREM-1) is a transmembrane receptor which is commonly found on innate immune cells, including neutrophils (29). Additionally, sTREM-1 plasma concentrations have strong mortality prediction in sepsis in Southeast Asia and are elevated in melioidosis (11, 30).

In our study an optimized cutoff of IL-1R2 assessed within 24 h of admission yielded both a reasonable sensitivity and specificity for 28-day mortality. While IL-1R2 protein measurements may not be widely available assays in clinical laboratories, our findings suggest that IL-1R2 alone or in combination with sTREM-1 could be adapted as a rapid point-of-care assay. Such tests, deployed to the bedside, could be used by a variety of personnel with varying levels of medical training, and may supplant the need for detailed clinical efforts to ascertain prognostic information.

Our study has a variety of strengths. This is one of the few reported studies to include prospectively enrolled patients with melioidosis in independent cohorts from different sites. All included subjects had culture-proven *B. pseudomallei* infection, the gold standard for melioidosis diagnosis. Both cohort studies had minimal missing data or loss to follow-up and thorough sample processing. Our model development and validation methodology was rigorous, followed TRIPOD guidelines, and also minimized bias effects and model overfitting. Finally, both cohorts utilized 28-day mortality (rather than hospital mortality) as the primary outcome, important in areas such as northeast Thailand where many critically ill patients are discharged at their request to die at home.

Our study also has several limitations. Both cohorts were drawn from referral healthcare centers in northeast Thailand and may not be representative outside those settings. In our derivation cohort, subjects were enrolled at the time of diagnosis, typically about 4 days after admission, whereas in our external validation cohort, subjects were enrolled within 24 h of hospital admission. These timing differences may account for the higher discrimination of IL-1R2 and sTREM-1 in our external validation cohort. Furthermore, we did not have a GCS score available in our derivation cohort, limiting complete assessments of clinical severity. Similarly, in both cohorts, the paucity of arterial blood gas and other data prevented complete SOFA calculations. Our plasma biomarker quantification of IL-1R2 utilized the same ELISA for both cohorts. However, our assessment of sTREM-1 used different ELISAs in each cohort, allowing for a possible difference in sensitivity and may explain the lower concentrations in the derivation cohort. ELISA methodology may also have lower sensitivity than other methods including bead-based multiplex and electrochemiluminescence assays. Furthermore, our analysis plan was designed *a priori* to select the simplest biomarker model while minimizing bias. However, other biomarkers such as CRP and PCT, which were not carried forward in our selection process, may furnish similar mortality discrimination to those analyzed. Finally, outside a randomized controlled trial, assessing the clinical utility of a biomarker model is challenging (31). Therefore, additional studies are required to better determine how our biomarkers might be adapted to a clinical context, including use in a point-of-care format.

In summary, we have developed and validated novel biomarker models with IL-1R2 protein for predicting 28-day mortality in patients with melioidosis. These models are a viable substitute for outcome prediction based on multiple clinical variables. If implemented in the future at the point-of-care in melioidosis-endemic areas, detection of IL-1R2 may be able to identify patients at highest risk of deterioration early in their course and inform clinical decision making.

## Data availability statement

The original contributions presented in the study are included in the article/Supplementary material, further inquiries can be directed to the corresponding author.

## Ethics statement

The studies involving human participants were reviewed and approved by the studies were approved by the ethics committee of Faculty of Tropical Medicine, Mahidol University (approval no. MUTM 2015-002-04, MUTM 2011-007-03, MUTM 2012-024-01, and MUTM 2018-047), Udon Thani Hospital Ethics Committee (00.32.102/318), Sunpasitthiprasong Hospital Ethics Committee (039/2556), the University of Washington Institutional Review Board (42988) and the Oxford University Tropical Research Ethics Committee (OXTREC172-12). Written informed consent to participate in this study was provided by the participants' legal guardian/next of kin.

## Author contributions

TK, NC, SW, and TW were involved in the design of the study. TK, TY, SW, LL-M, GR, DD, and AD collected samples and performed experiments. RP, VH, and GL recruited patients and collected data. TW, SW, and TK performed the statistical analysis. ND, NC, DL, and GL provided facilities. NC and TW were responsible for supervision of the study. All authors were involved in writing the paper and have approved the final version.

## Funding

This work was supported by the United States National Institutes of Health [grant numbers T32GM086270, K12HD047349, K08HL157562, R01HL113382, R01AI137111, and U01AI115520] and the Wellcome Trust [090219/Z/09/Z; 101103/Z/13/Z; 220211]. The content is solely the responsibility of the authors and does not necessarily represent the official views of the National Institutes of Health or the Wellcome Trust. For the purpose of Open Access, the authors have applied a CC BY public copyright license to any author accepted manuscript version arising from this submission.

## Conflict of interest

The authors declare that the research was conducted in the absence of any commercial or financial relationships that could be construed as a potential conflict of interest.

## Publisher’s note

All claims expressed in this article are solely those of the authors and do not necessarily represent those of their affiliated organizations, or those of the publisher, the editors and the reviewers. Any product that may be evaluated in this article, or claim that may be made by its manufacturer, is not guaranteed or endorsed by the publisher.
