# Supplementary material for: IL-1R2-based biomarker models predict melioidosis mortality independent of clinical data
Source: Front Med (Lausanne). 2023 Jun 29;10:1211265. doi: 10.3389/fmed.2023.1211265 (PMC10338910; doi:10.3389/fmed.2023.1211265)
Supplement: Supplementary file 1 [file Data_Sheet_1.docx]

***Supplementary Material***

**IL-1R2-based biomarker models predict melioidosis mortality independent of clinical data**

**Taniya Kaewarpai^1^, Shelton W. Wright^2^, Thatcha Yimthin^1^, Rungnapa Phunpang^3^, Adul Dulsuk^4^, Lara Lovelace-Macon^5^, Guilhem F. Rerolle^5^, Denisse B. Dow^5^, Viriya Hantrakun^3^, Nicholas P.J. Day^3,9^, Ganjana Lertmemongkolchai^7,8^, Direk Limmathurotsakul^3,6^, T. Eoin West ^5,10†^ , Narisara Chantratita^1,3†*^**

*** Correspondence:** Narisara Chantratita: [narisara@tropmedres.ac](mailto:narisara@tropmedres.ac)

***Supplementary Figures and tables***

**Supplementary Figure 1. Study Profiles: Analysis flow diagrams of the derivation and external validation cohorts**

**Supplementary Figure 2. Net benefit of the IL-1R2+sTREM-1 model compared to the IL-1R2 model in the external validation cohort**

**Supplementary Table S1. Biomarker model assessments in the derivation and external validation cohorts**

**Supplementary Table S2. IL-1R2 model improvement with addition of sTREM-1 in the derivation cohort**

**Supplementary Table S3. Biomarkers by 28-day survival status of melioidosis patients in the external validation cohort**

**Supplementary Table S4. Net benefit of adding sTREM-1 to the IL-1R2 model for mortality prediction in the external validation cohort**

**Supplementary Table S5. Clinical performance of an IL-1R2 score in the external validation cohort**

**Supplementary Figure 1. Study Profiles: Analysis flow diagrams of the derivation and external validation cohorts**


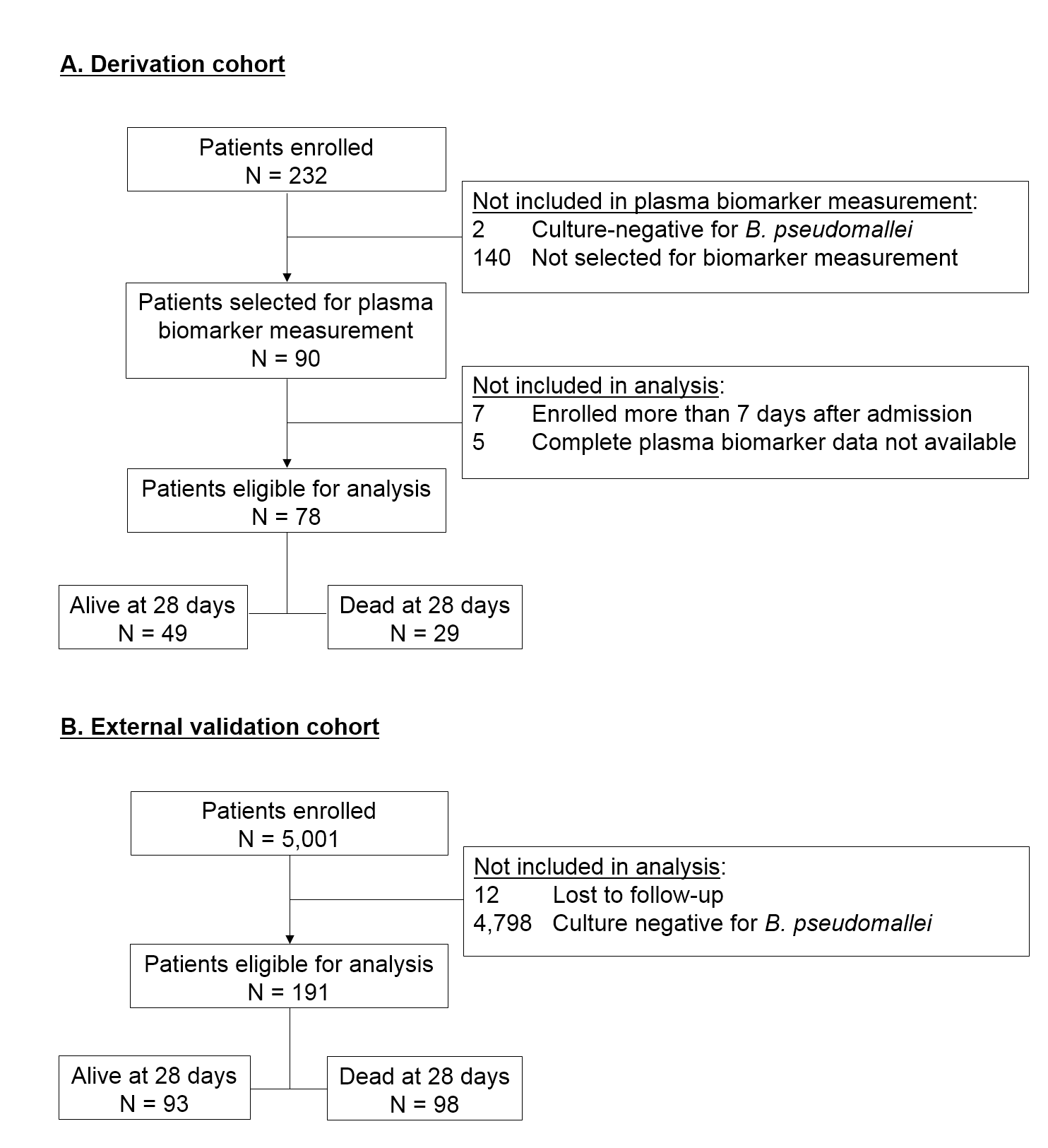



**Supplementary Figure 2. Net benefit of the IL-1R2+sTREM-1 model compared to the IL-1R2 model in the external validation cohort.** The graph represents the net benefit of the IL-1R2 + sTREM-1 model (solid black line) compared to the IL-1R2-only model (dashed gray line). The dashed black line represents the assumption that all patients will die within 28 days. The solid gray line represents the assumption that no patients will die within 28 days. The net benefit is derived from the equation ((true positive count/N)-(false positive count/N)*(threshold probability/1-threshold probability)). Threshold probability, on the x-axis, occurs when the expected benefit of using a model is equal to the expected benefit of not using a model. At the threshold probability of 0, assuming all patients die ("All die") yields a net benefit of 0.51 (28-day mortality in the external validation cohort). The net benefit of 0.51 would be equivalent to correctly identifying 51 patients who would die per 100 patients. Assuming no patients will die ("None die") gives a net benefit of 0 for the entire range of threshold probabilities. "All die" crosses with "None die" at the threshold probability of 0.51 (again, the 28-day mortality in the external validation cohort) and continues to a negative net benefit at higher threshold probabilities. For each score, the predicted mortality of the model was used in net benefit estimations.

**Supplementary Table S1. Biomarker model assessments in the derivation and external validation cohorts**

| **Model** | **H-Lχ^2^/P value^a^** | **Optimism-corrected AUC (95% CI)^b^** |
| --- | --- | --- |
| **Derivation cohort** |  |  |
| **Organ failure** | 18.3/0.01 | 0.64 (0.50-0.76) |
| **IL-1R2 + sTREM-1** | 11.1/0.20 | 0.81 (0.71-0.90) |
| **IL-1R2** | 11.1/0.20 | 0.78 (0.67-0.87) |
| **sTREM-1** | 2.4/0.97 | 0.62 (0.46-0.74) |
| **External validation cohort** |  |  |
| **Modified SOFA** | 7.52/0.38 | 0.77 (0.69-0.84) |
| **IL-1R2 + sTREM-1** | 2.7/0.95 | 0.86 (0.81-0.91) |
| **IL-1R2** | 5.9/0.66 | 0.85 (0.79-0.91) |
| **sTREM-1** | 1.6/0.98 | 0.80 (0.73-0.87) |

^a^ Hosmer-Lemeshow goodness of fit χ^2^ & P value

^b^ Optimism-correction based on 1000 set bootstrap replication

**Supplementary Table S2. IL-1R2 model improvement with addition of sTREM-1 in the derivation cohort**

| Model | Variable | Crude OR | 95% CI | P value | LR^a^ | IDI^b^ |
| --- | --- | --- | --- | --- | --- | --- |
| **IL-1R2** | IL-1R2 | 33.4 | 5.8-192.8 | <0.001 | ref | ref |
| **IL-1R2 + sTREM-1** | IL-1R2 | 39.8 | 6.4-247.6 | <0.001 | 0.02 | 0.06 ± 0.03,  P=0.05 |
|  | sTREM-1 | 4.9 | 1.1-21.0 | 0.04 |  |  |

^a^ P values derived from likelihood ratio tests comparing the models of the IL-1R2 + sTREM-1 model to the model of IL-1R2 alone

^b^ IDI: Integrated discrimination improvement; values represent model improvement estimates for models of IL-1R2 + sTREM-1 over an IL-1R2 model alone ± standard error

**Supplementary Table S3. Biomarkers by 28-day survival status of melioidosis patients in the external validation cohort**

| **Biomarker (pg/ml): median (IQR)** | **All**  **(N=191)** | **Survivors**  **(N=49)** | **Non-survivors (N=98)** | **P value** |
| --- | --- | --- | --- | --- |
| **IL-1R2** | 132847 (51363-393382) | 58452 (38516-108615) | 317817 (157672-495432) | <0.001 |
| **sTREM-1** | 419 (92-979) | 141 (92-411) | 783 (411-1303) | <0.001 |

**Supplementary Table S4. Net benefit of adding sTREM-1 to the IL-1R2 model for mortality prediction in the external validation cohort**

| **Probability of death**  **(%)** | **Model net benefit** | | | |  | **Advantage of adding sTREM-1 to the IL-1R2 model** | |
| --- | --- | --- | --- | --- | --- | --- | --- |
|  | **Predict all die** | **IL-1R2** | **IL-1R2 +**  **sTREM-1** | **Advantage of adding sTREM-1** |  | **Increase in # correctly predicted to die per 100 patients** | **Reduction in # incorrectly predicted to die per 100 patients** |
| 10 | 0.46 | 0.45 | 0.46 | 0.0105 |  | 1.0 | 9.4 |
| 20 | 0.39 | 0.41 | 0.43 | 0.0183 |  | 1.8 | 7.3 |
| 30 | 0.30 | 0.38 | 0.39 | 0.0142 |  | 1.4 | 3.3 |
| 40 | 0.19 | 0.33 | 0.35 | 0.0227 |  | 2.3 | 3.4 |
| 50 | 0.03 | 0.31 | 0.32 | 0.0105 |  | 1.0 | 1.0 |

**Supplementary Table S5. Clinical performance of an IL-1R2 score in the external validation cohort**

| **Performance (95% CI)** | **IL-1R2 concentration**  **(pg/ml)** |
| --- | --- |
|  | **≥ 173911** |
| **Sensitivity** | 72 (63-81) |
| **Specificity** | 85 (76-92) |
| **Positive Predictive Value** | 84 (74-91) |
| **Negative Predictive Value** | 75 (65-83) |
| **Positive Likelihood Ratio** | 4.8 (2.9-7.9) |
| **Negative Likelihood Ratio** | 0.3 (0.2-0.5) |
